# Supplementary material for: Maternal Plasma miRNAs as Early Biomarkers of Moderate-to-Late-Preterm Birth
Source: Int J Mol Sci. 2024 Sep 2;25(17):9536. doi: 10.3390/ijms25179536 (PMC11394737; doi:10.3390/ijms25179536)
Supplement: Supplementary file 1 [file ijms-25-09536-s001.zip › ijms-3131250-supplementary.pdf]

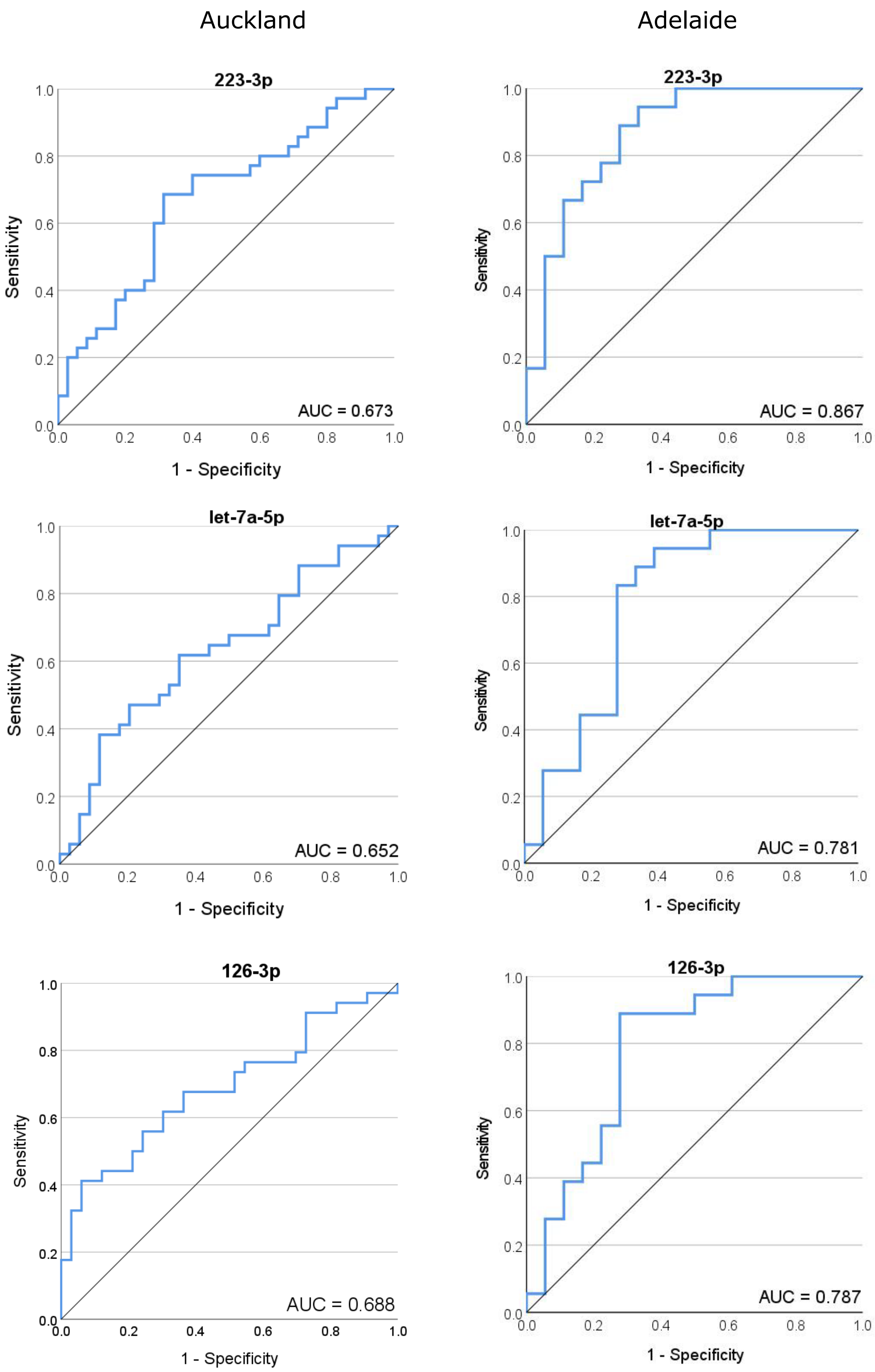

Supplementary Figure S1.

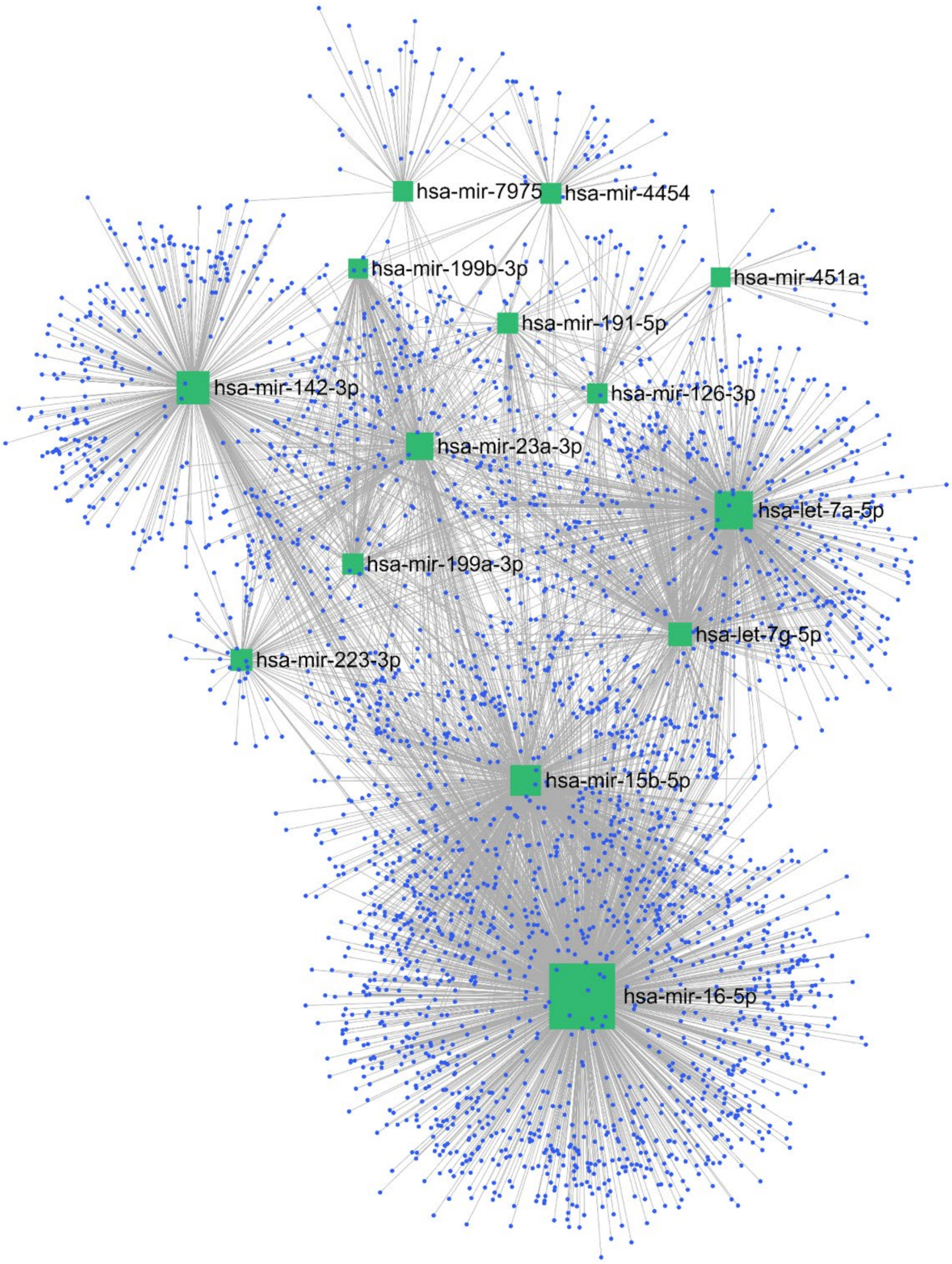

Supplementary Figure S2.

Supplementary Table S1:

**Pathways**	**Total Genes**	**Expected**	**Hits**	**Pval**	**FDR**
cell-substrate adhesion	241	3.5	15	2.1E-06	0.0001
cell-matrix adhesion	159	2.3	12	3.1E-06	0.0001
regulation of cell cycle	886	12.7	31	3.5E-06	0.0001
cell cycle arrest	428	6.1	19	1.3E-05	0.0003
regulation of transferase activity	768	11.0	27	1.5E-05	0.0003
negative regulation of cell cycle	520	7.5	21	1.9E-05	0.0003
regulation of kinase activity	743	10.7	26	2.3E-05	0.0003
positive regulation of cell migration	263	3.8	14	2.7E-05	0.0003
protein phosphorylation	1480	21.3	41	2.9E-05	0.0003
regulation of protein kinase activity	698	10.0	24	6.5E-05	0.0006
regulation of gene expression, epigenetic	155	2.2	10	8.0E-05	0.0006
gene silencing	99	1.4	8	8.8E-05	0.0006
regulation of cell adhesion	294	4.2	14	9.0E-05	0.0006
phosphorylation	1680	24.1	43	1.0E-04	0.0006
cell proliferation	1900	27.3	47	1.1E-04	0.0006
regulation of translation	228	3.3	12	1.1E-04	0.0006
protein import into nucleus	228	3.3	12	1.1E-04	0.0006
gland development	303	4.4	14	1.2E-04	0.0006
homeostasis of number of cells	196	2.8	11	1.2E-04	0.0006
regulation of phosphorylation	1070	15.3	31	1.3E-04	0.0006
regulation of cell proliferation	1430	20.5	38	1.3E-04	0.0006
cell division	507	7.3	19	1.3E-04	0.0006
nuclear import	232	3.3	12	1.3E-04	0.0006
response to drug	344	4.9	15	1.3E-04	0.0006
growth	839	12.0	26	1.7E-04	0.0007
regulation of protein phosphorylation	987	14.2	29	1.8E-04	0.0007
regulation of cellular protein metabolic process	1560	22.4	40	1.8E-04	0.0007
morphogenesis of an epithelium	440	6.3	17	2.1E-04	0.0007
cell development	1840	26.5	45	2.1E-04	0.0007
cell cycle checkpoint	281	4.0	13	2.1E-04	0.0007
cellular response to stress	1620	23.3	41	2.1E-04	0.0007
G1/S transition of mitotic cell cycle	209	3.0	11	2.2E-04	0.0007
regulation of molecular function	2250	32.3	52	2.3E-04	0.0007
reproductive process	1740	25.0	43	2.4E-04	0.0007
reproduction	1860	26.6	45	2.4E-04	0.0007

immune system development	722	10.4	23	2.8E-04	0.0007
regulation of cyclin-dependent protein kinase activity	89	1.3	7	2.9E-04	0.0007
negative regulation of cell differentiation	540	7.8	19	2.9E-04	0.0007
negative regulation of apoptotic process	679	9.7	22	3.1E-04	0.0007
hematopoietic or lymphoid organ development	679	9.7	22	3.1E-04	0.0007
negative regulation of apoptotic process	679	9.7	22	3.1E-04	0.0007
regulation of cell migration	456	6.5	17	3.1E-04	0.0007
response to abiotic stimulus	876	12.6	26	3.4E-04	0.0008
regulation of cell differentiation	1290	18.5	34	3.6E-04	0.0008
viral reproductive process	597	8.6	20	3.8E-04	0.0008
negative regulation of programmed cell death	691	9.9	22	3.9E-04	0.0008
focal adhesion assembly	44	0.6	5	4.0E-04	0.0008
regulation of catalytic activity	1730	24.9	42	4.1E-04	0.0009
wound healing	700	10.0	22	4.6E-04	0.0009
positive regulation of cell adhesion	127	1.8	8	4.9E-04	0.0010
tissue morphogenesis	566	8.1	19	5.2E-04	0.0010
intracellular receptor mediated signaling pathway	270	3.9	12	5.3E-04	0.0010
nuclear transport	392	5.6	15	5.4E-04	0.0010
G1 phase of mitotic cell cycle	47	0.7	5	5.4E-04	0.0010
programmed cell death	2160	30.9	49	5.6E-04	0.0010
protein import	272	3.9	12	5.7E-04	0.0010
G1 phase	49	0.7	5	6.6E-04	0.0012
epidermal growth factor receptor signaling pathway	167	2.4	9	6.9E-04	0.0012
negative regulation of cell proliferation	585	8.4	19	7.7E-04	0.0013
apoptotic process	2130	30.5	48	7.7E-04	0.0013
apoptotic process	2130	30.5	48	7.7E-04	0.0013
regulation of protein modification process	1250	17.9	32	8.9E-04	0.0014
hemopoiesis	640	9.2	20	9.1E-04	0.0014
positive regulation of cell proliferation	786	11.3	23	9.2E-04	0.0014
behavior	597	8.6	19	9.8E-04	0.0015
ER-nucleus signaling pathway	111	1.6	7	1.1E-03	0.0017
response to UV	112	1.6	7	1.2E-03	0.0017
response to ionizing radiation	112	1.6	7	1.2E-03	0.0017

regulation of MAPK cascade	559	8.0	18	1.2E-03	0.0017
myeloid cell differentiation	296	4.3	12	1.2E-03	0.0017
apoptotic mitochondrial changes	83	1.2	6	1.2E-03	0.0017
blood coagulation	564	8.1	18	1.3E-03	0.0018
regulation of programmed cell death	1550	22.3	37	1.3E-03	0.0018
regulation of gene expression	4480	64.3	85	1.4E-03	0.0018
coagulation	568	8.2	18	1.4E-03	0.0018
MAPK cascade	665	9.5	20	1.4E-03	0.0018
nucleocytoplasmic transport	388	5.6	14	1.5E-03	0.0018
response to radiation	345	5.0	13	1.5E-03	0.0018
hemostasis	570	8.2	18	1.5E-03	0.0018
cellular component assembly	1790	25.7	41	1.5E-03	0.0019
interphase of mitotic cell cycle	435	6.2	15	1.6E-03	0.0019
negative regulation of developmental process	674	9.7	20	1.7E-03	0.0020
regulation of mitotic cell cycle	351	5.0	13	1.7E-03	0.0020
positive regulation of epithelial cell proliferation	120	1.7	7	1.7E-03	0.0020
cell cycle	1860	26.7	42	1.7E-03	0.0020
insulin receptor signaling pathway	192	2.8	9	1.8E-03	0.0021
generation of neurons	1300	18.7	32	1.8E-03	0.0021
interphase	443	6.4	15	1.9E-03	0.0021
regulation of body fluid levels	680	9.8	20	1.9E-03	0.0021
regulation of apoptotic process	1540	22.0	36	2.0E-03	0.0023
regulation of protein metabolic process	1820	26.2	41	2.2E-03	0.0024
neuron development	945	13.6	25	2.2E-03	0.0024
protein import into nucleus, translocation	39	0.6	4	2.3E-03	0.0025
response to oxidative stress	279	4.0	11	2.4E-03	0.0025
positive regulation of transcription from RNA polymerase II promoter	800	11.5	22	2.6E-03	0.0027
response to hormone stimulus	751	10.8	21	2.7E-03	0.0028
neurogenesis	1390	20.0	33	2.7E-03	0.0028
viral reproduction	803	11.5	22	2.7E-03	0.0028
positive regulation of transferase activity	510	7.3	16	2.8E-03	0.0029
macromolecule modification	3430	49.2	67	2.9E-03	0.0029
